# Dosimetric comparisons of VMAT, IMRT and 3DCRT for locally advanced rectal cancer with simultaneous integrated boost

**DOI:** 10.18632/oncotarget.6401

**Published:** 2015-11-26

**Authors:** Jun Zhao, Weigang Hu, Gang Cai, Jiazhou Wang, Jiang Xie, Jiayuan Peng, Zhen Zhang

**Affiliations:** ^1^ Department of Radiation Oncology, Fudan University Shanghai Cancer Center and Department of Oncology, Shanghai Medical College, Fudan University, Shanghai 200032, China

**Keywords:** rectal radiotherapy, VMAT, dose comparison

## Abstract

The simultaneous integrated boost radiotherapy for preoperative locally advanced rectal cancer (LARC) can improve the local control and overall survival rates. The purpose of this study is to compare the dosimetric differences among volumetric modulated arc therapy (VMAT), fixed-field intensity modulated radiotherapy (IMRT) and three-dimensional conformal radiotherapy (3DCRT) for the LARC. Ten LARC patients treated in our department using the simultaneous escalate strategy were retrospectively analyzed in this study. All patients had T3 with N+/− and were treated with IMRT. Two additional VMAT and 3DCRT plans were created for each patient. VMAT plans were designed using SmartArc planning module. Both IMRT and SmartArc had similar optimization objectives. The prescription was 50 Gy to the planning clinical target volume (PTV-C) and 56 Gy to the planning gross target volume (PTV-G). The target coverage and organs at risk (OARs) were compared for all the techniques. The paired, two-tailed Wilcoxon signed-rank test was applied for statistical analysis. Results of this study indicate that IMRT and SmartArc were all significantly superior to 3DCRT in most of the relevant values evaluated of target response, OARs and normal tissue sparing. They provided comparable dosimetric parameters for target volume. But IMRT shows better sparing for OARs and normal tissue.

## INTRODUCTION

Locally advanced rectal cancer (LARC) is a common oncological diagnosis in our country and preoperative radiochemotherapy is the standard neoadjuvant treatment. Previous studies have shown that the preoperative radiochemotherapy provided significantly lower local recurrence rate and less acute and chronic toxicity as compared to postoperative radiochemotherapy [1–7]. A preoperative therapy trial published recently has revealed a significant 5-year disease-free survival rate improvement [8]. However, the acute and chronic small bowel and rectal toxicities are common causes of morbidity during radiochemotherapy for rectal cancer [9]. The severe toxicity may limit the further dose escalation or lead to prolonged treatment interruptions or premature termination of the radiation course, which may reduce the therapy effectiveness.

Several groups have studied the dose-volume relationship between the amount of small bowel receiving intermediate- and low-doses of radiation and the rates of severe diarrhea [10–13]. They found that a strong dose–volume relationship existed for the development of Grade 3 acute small bowel toxicity in patients receiving preoperative radiochemotherapy. Therefore, there has been great interest in the application of highly conformal treatment approaches, such as IMRT and VMAT, for producing highly conformal dose distributions in the target volumes and minimizing the dose to OARs. Several planning studies have done for the LARC by using different treatment approaches, such as proton therapy, VMAT, IMRT and 3DCRT [14–18]. However, only few studied the dosimetric difference among these techniques in the treatment of simultaneous integrated boost radiotherapy for preoperative locally advanced rectal cancer [19]. In ref. [19], the results show that VMAT plans can provide similar sparing of OARs to IMRT and have higher efficiency. However, according to our clinical experience, IMRT plans can achieve better sparing of OARs with similar target coverage when compare to VMAT plans.

The purpose of this study is to compare the OARs sparing without compromising the target coverage among VMAT, IMRT and 3DCRT plans in 10 patients. Target coverage and target dose distribution, comformality, normal tissue avoidance, and irradiated body volume were evaluated and compared for different plans.

## RESULTS

**Figure 1:**
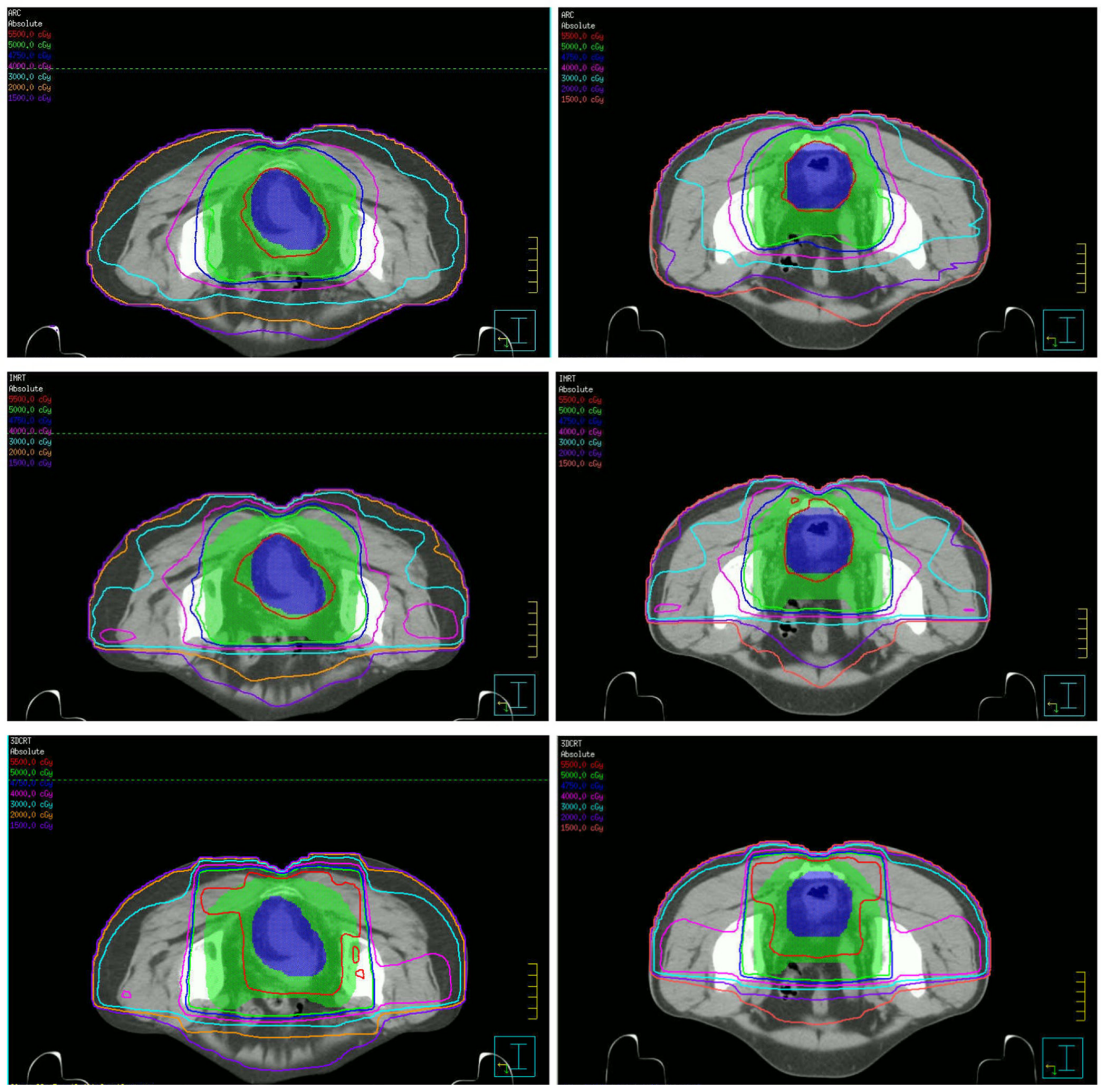
Dose distributions of three planning techniques for two patients in the axial slices From up to down are SmartARC, IMRT and 3DCRT respectively.

**Figure 2:**
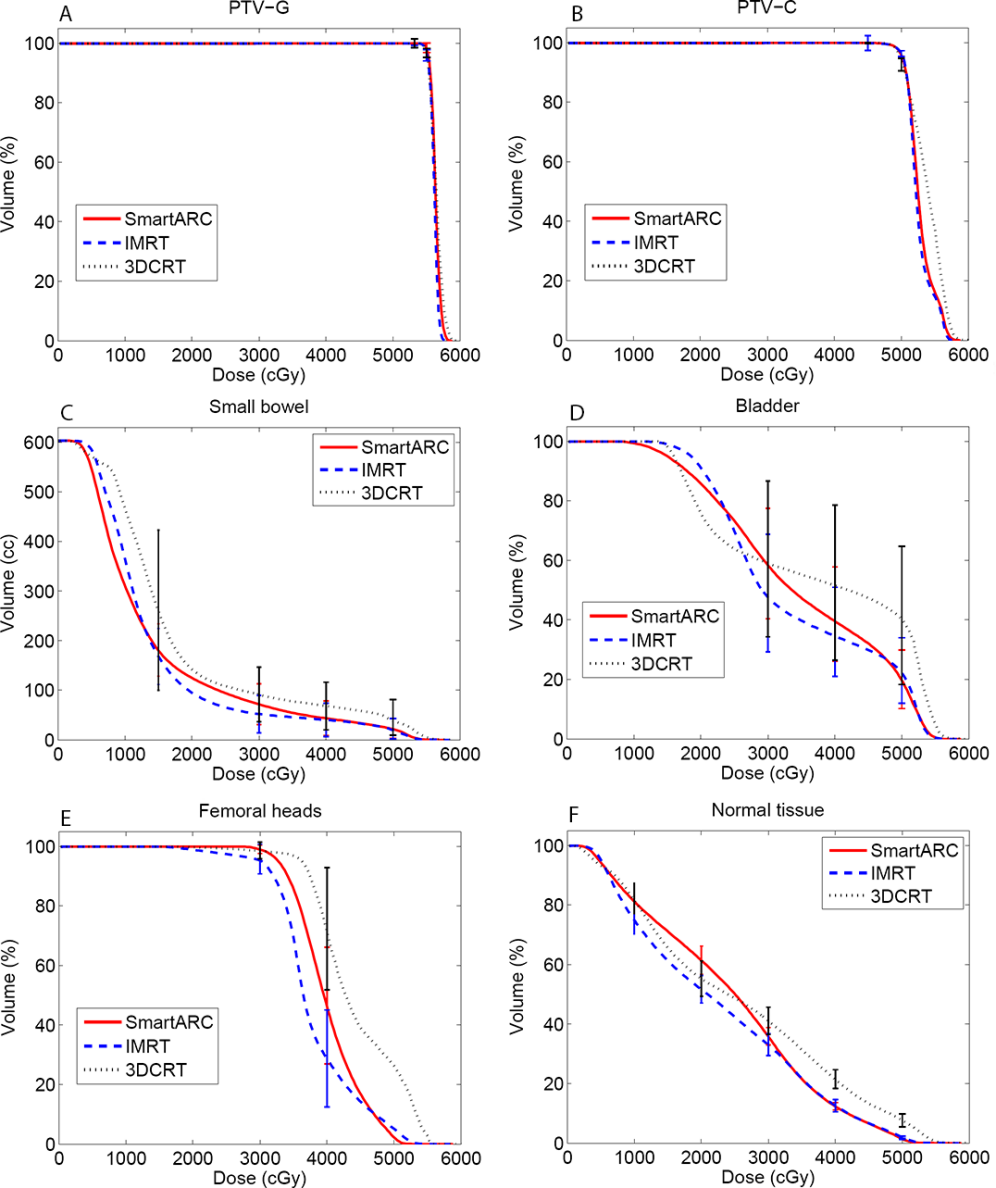
Cumulative DVH of the target (A: PTV-G; B: PTV-C), the OARs (C: Small bowel; D: Bladder; E: Femoral heads) and normal tissue (F: Normal tissue) of the three treatment planning techniques.

The dose distributions for the three different treatment plans including 3DCRT, IMRT and SmartArc in the axial slices of two patients were shown in the Figure 1. The isodoses were set from 15–55 Gy. The PTV-C was outlined as a green area in all images and the PTV-G was shown as the blue area. The average cumulative DVHs of the ten patients for the PTV-C, the PTV-G, the OARs (bladder, small bowel and femoral heads) and normal tissues were calculated and plotted in Figure 2. The statistical dosimetric evaluation and comparison of the three planning techniques were listed in Table 1.

**Table 1:** Dose statistics comparison for planning target volumes and organs at risk

Parameter	SmartARC	IMRT	3DCRT	SmartARC vs IMRT	SmartARC vs 3DCRT	IMRT vs 3DCRT
***PTV-G**. Volume (cm^3^):247.6 ± 54.6 (156.2−340.3)*						
D1% (Gy)	57.4 ± 0.6	57.1 ± 0.3	58.3 ± 0.5	*n*	0.005	0.005
D99% (Gy)	55 ± 0.3	54.8 ± 0.4	55.4 ± 0.2	*n*	*n*	*n*
mean (Gy)	56.5 ± 0.6	56.1 ± 0.2	56.4 ± 0.3	0.037*	*n*	0.013
V95% (%)	100.0 ± 0.1	100.3 ± 1.1	100.0 ± 0.1	*n*	*n*	*n*
V100% (%)	98.5 ± 1.6	96.8 ± 1.9	98.9 ± 1.5	0.007	*n*	*n*
HI	0.0 ± 1	0.0 ± 0.0	0.0 ± 0.0	*n*	*n*	0.003
CI	1.2 ± 0.2	1.1 ± 0.1	3.1 ± 0.6	0.028*	0.005	0.005
***PTV-C**. Volume (cm^3^): 1844.8 ± 215.2 (1562.3–2310.8)*						
D99% (Gy)	48.8 ± .4	48.7 ± 0.4	48.9 ± 0.6	*n*	*n*	*n*
mean (Gy)	52.8 ± 0.3	52.6 ± 0.4	53.9 ± 0.3	*n*	0.005	0.005
V95% (%)	100.0 ± 0.1	100.0 ± 2.5	100.0 ± 0.2	*n*	*n*	*n*
V100% (%)	95.0 ± 0.0	96.5 ± 1.0	94.7 ± 2.1	*n*	*n*	*n*
CI	1.0 ± 0.3	1.1 ± 0.2	1.4 ± 0.1	0.005	0.005	0.005
***Small bowel**. Volume (cm^3^):683.7 ± 330.4 (406.2–1488.5*						
mean (Gy)	15.2 ± 3.2	15.1 ± 3.1	19.2 ± 0.6	*n*	0.005	0.005
V15 (cc)	180.9 ± 53.7	167.9 ± 56.4	261.6 ± 161.5	*n*	0.047	0.013
V30 (cc)	71.6 ± 41.1	51.9 ± 37.7	91.1 ± 55.4	0.005*	0.022	0.005
V40 (cc)	43.3 ± 35.5	40.2 + 33.8	68.4 ± 48.1	*n*	0.005	0.005
V50 (cc)	22.4 ± 20.0	22.5 ± 35.8	44.9 ± 35.8	*n*	0.005	0.005
***Bladder**. Volume (cm^3^): 117.4 ± 76.7 (50.0–259.7*						
mean (Gy)	36.9 ± 5.6	36.0 ± 5.3	40.0 ± 8.7	0.017*	*n*	0.022
V_30_ (%)	64.7 ± 18.6	54.6 ± 19.8	67.3 ± 26.2	0.005*	*n*	0.009
V_40_ (%)	44.2 ± 15.8	38.9 ± 15.0	59.2 ± 26.1	0.005*	0.013	0.005
V_50_ (%)	22.2 ± 9.8	25.0 ± 11.0	45.7 ± 23.2	0.047	0.005	0.005
***Femoral heads**. Volume (cm^3^): 94.4 ± 20.2 (68.8–121.4)*						
mean (Gy)	40.4 ± 2.2	38.3 ± 2.7	44.6 ± 3.4	0.005*	0.005	0.005
V_30_ (%)	99.1 ± 1.4	95.8 ± 5.0	98.7 ± 2.8	0.012*	*n*	0.012
V_40_ (%)	49.3 ± 18.6	31.5 ± 16.3	71.1 ± 20.5	0.005*	0.007	0.005
***Normal tissue**. Volume (cm^3^): 9956.4 ± 1877.9 (7308.3–11879.9)*						
mean (Gy)	24.5 ± 1.2	22.8 ± 1.5	25.9 ± 1.9	0.005*	0.017	0.005
V_10_ (cc)	8119.8 ± 1222.0	7979.4 ± 1067.9	8155.5 ± 1154.7	0.005*	*n*	0.015
V_20_ (cc)	6141.0 ± 818.4	5154.6 ± 639.4	5520.9 ± 645.3	0.005*	0.007*	0.009
V_30_ (cc)	3574.0 ± 484.8	3296.0 ± 371.0	4101.4 ± 588.5	0.013*	0.007	0.005
V_40_ (cc)	1232.7 ± 154.8	1230.0 ± 103.0	2131.9 ± 458.8	*n*	0.005	0.005
V_50_ (cc)	123.5 ± 24.9	199.3 ± 28.2	763.4 ± 150.8	0.005	0.005	0.005

Dosimetric comparison of three treatment planning techniques. *p* value normally displayed means the former is better than the latter. *p* value displayed with a star superscript means the latter is better than the former. *p* value of *n* means no statistically significant.

### Target coverage and dose distribution

All three techniques met the clinical requirements. For PTV-G, IMRT exhibited better homogeneity index (HI) than 3DCRT (*p* = 0.003) and both SmartARC and IMRT exhibited better conformality index (CI) than 3DCRT with same *p* value (both *p* = 0.005). There are no HI differences for PTV-G when compare SmartARC with IMRT and 3DCRT (*p* = 0.598 and *p* = 0.097 respectively). IMRT had superior CI to SmartArc (*p* = 0.028).

For PTV-C, both SmartARC and IMRT exhibited better CI than 3DCRT with same *p* value (both *p* = 0.005). SmartARC had superior CI to IMRT (*p* = 0.005).

### Small bowel and bladder

The mean volumes of small bowel and bladder in this study were 683.7 cm^3^ (ranged from 406.2 to 1488.5 cm^3^) and 117.4 cm^3^ (ranged from 50.0 to 259.7 cm^3^), respectively. Compared to the 3DCRT, the IMRT and SmartArc showed significant sparing improvement for almost all the evaluated dosimetric parameters. For the small bowel, there were no significant differences between the IMRT and SmartArc on V15, V40, V50 and D_mean._ Although the V30 was lower for IMRT than SmartArc, a slightly higher dose for low-dose area (< 12 Gy) in IMRT was observed (Figure 2C). For the bladder, the IMRT showed superior dosimetric results to SmartArc on V_30_, V_40_, and D_mean._ Again, the IMRT had slight higher dose on the low-dose area and a slight higher V_50._

### Femoral heads

The volume of femoral heads ranged from 68.8 to 121.4 cm^3^ with a mean value of 94.4 cm^3^ Similar to the bladder, the IMRT and SmartArc had superior sparing to the 3DCRT. However, compared to the SmartArc, the IMRT demonstrated statistically significant benefit with lower dose on the femoral heads (all *p* < 0.05). This could be also found in the related DVH (Figure 2E).

### Normal tissue

IMRT had superior normal tissue sparing to 3DCRT for all the values compared. SmartArc also had superior normal tissue sparing to 3DCRT except the V_10_ and V_20_. They were equivalent on the V_10_ but SmartArc was worse on the V_20_. IMRT had better sparing on the V_10_, V_20_, V_30_, and D_mean_ than SmartArc in the normal tissue and they were equivalent on the V_40_. However, the SmartArc had the best advantage of reducing V_50_.

## DISCUSSION

This dosimetric study compared the dose variability among the VMAT, IMRT, 3DCRT for the simultaneously integrated boost rectal cancer radiotherapy. A few studies have investigated different treatment techniques (3DCRT, IMRT, VMAT and Proton) for rectal cancer [14, 17, 18, 19]. However, only one study was concerned on the SIB radiotherapy [19].

When comparing SmartArc and IMRT to 3DCRT technique, all the results revealed obvious superiority of the target coverage and OARs sparing. SmartArc and IMRT achieved comparable results in most of the evaluated endpoints on the target. Such as PTV-G, IMRT plans got lower mean dose and better conformity index. However, VMAT plans resulted in better conformality for the PTV-C volume. With regard to OARs sparing, IMRT plans was significantly superior to SmartArc in most of the relevant values for bladder, femoral heads and normal tissue surrounding target volume. But for small bowel, the IMRT and SmartArc were comparable except for V_30._ Additionally, IMRT plans produced significant volume sparing from 30 Gy isodose line.

For the rectal radiochemotherapy, acute gastrointestinal toxicity (notably diarrhea) is one of the most common complications. Previous studies have demonstrated a strong dose –volume relationship between the severities of diarrheal toxicity and irradiated small bowel volume at different dose levels [10–13]. Baglan KL et al. indicated a strong dose–volume relationship existing between the irradiated small bowel volume and acute diarrhea at all dose levels and they constructed a predictive model for acute toxicity [10]. Subsequent studies have confirmed the significance of intermediate dose levels V_15_, V_20_ and V_25_ with respect to severe diarrhea [12, 13]. From the cumulative DVH of small bowel in our study, it is clear that IMRT reveals significant lower irradiated volume than SmartArc and 3DCRT in the intermediate dose range (15–35 Gy). This indicates that IMRT plans will lower the risk of acute toxicity for small bowel after radiochemotherapy.

In summary, our results revealed the IMRT technique as the best technique for current study. Although IMRT and SmartArc achieved the comparable target coverage, IMRT was better in OARs and normal tissue sparing. And obviously, IMRT and SmartArc were superior to 3DCRT in most clinically evaluated endpoints.

## MATERIALS AND METHODS

### Patient selection and imaging

Ten consecutive patients of locally advanced rectal cancer with simultaneous integrated boost treated with preoperative radiochemotherapy at our department were involved in this study. All ten patients had T3 stage with N0 (1 patient) and N1–2 (9 patients). None of them had evidence of distant metastasis (M0). The median age was 56.9 years (ranged from 32 to 65). All patients were immobilized, simulated and treated in the prone position using a carbon-fiber belly board apparatus to achieve abdominal contents avoidance. The planning CT was scanned in a big core CT (Philips Brilliance CT) with slice thickness of 5 mm. No specific bladder filling or empty instructions were given to patients. All procedures were passed through the ethical standards of our institute.

### Target delineation and treatment planning

Target volumes and organs at risk were delineated on the Pinnacle^3^ treatment planning system (TPS, Philips Medical Systems, Pinnacle v9.0, Milpitas, CA). The gross tumor volume (GTV) was determined by a combination of findings on physical exam, CT, MRI, and/or PET-CT. The planning target volume PTV-G was GTV plus a 12 mm uniform margin. The clinical target volume (CTV) included the GTV, the internal iliac, pre-sacral and peri-rectal nodal groups, the external iliac nodal region (if lesions extended into gynecologic/genitourinary structures or positive external iliac lymph nodes) and the inguinal nodal region (if lesions extended to the anal verge, peri-anal skin or positive inguinal nodes). The PTV-C was generated with a 8–10 mm asymmetrical margin around the CTV. A 8-mm margin was used in areas where the lesions were close to the small bowel, bladder and femoral heads, while a 10-mm margin was used elsewhere. The small bowel, bladder and femoral heads were defined as organs at risk.

The prescribed doses were 50 Gy to the PTV-C volume and 56 Gy simultaneous to the PTV-G volume in 25 treatment fractions. The plans were optimized to meet the following criteria: less than 50% of bladder volume received 40Gy and no volume should receive 60 Gy; less than 20 cc of small bowel received 50 Gy and less than 100 cc received 40 Gy; less than 25% of femoral heads received 45 Gy.

All patients were treated with IMRT technique on the Trilogy linac with 6 MV photons and Millennium 120–leaf MLC (Varian Medical Systems, Palo Alto, CA).

IMRT plans were created in the Pinnacle^3^ TPS using the direct machine parameter optimization (DMPO) with a total segment of 50. For the dosimetric comparison purpose, a 3DCRT and a VMAT plan were additionally created to each patient and compared to the actually delivered IMRT plan. During the 3DCRT planning, beam angles were both set to 0°, 90° and 270° to the PTV-C and PTV-G with different beam shapes. This beam arrangement helped to avoid the direct irradiation on the small bowel. The 6 MV was used for beam 0° and 15 MV for the others. Beam weightings and wedges (40° or 45°) were manually adjusted for each patient. VMAT plans were designed using SmartArc planning module. Dual 6-MV partial arcs were used in our planning (clockwise from 220° to 140°CW and counterclockwise from 140° to 220°) to avoid beams incidence from the front of patient abdomen. The final dose calculation was performed using a 4-degree spacing with a total of 142 control points. Collimator angle was set to 15° to minimize the tongue-and-groove effect. The dose rate of Varian Trilogy varied range from 0 μ/min to a maximum of 600 μ/min.

### Dosimetric comparisons and statistical analysis

Dosimetric evaluation of all plans was performed using dose volume histogram (DVH). For target coverage, the mean dose (D_mean_), D1% (dose to _1%_ of the volume) and D_99%_ (dose to 99% of the volume), V_95%_ (volume of the target receiving at least 95% of the prescribed dose) and V100% to the PTV-G and PTV-C were investigated. The homogeneity of the plans was measured in terms of the homogeneity index, which was expressed as (D_5%_–D_95%_)/prescribed dose. The conformality of the plans was also evaluated with a conformality index defined as the ratio of the target volume receiving 95% of the prescribed dose divided by the total volume receiving that dose level.

Organs at risk were evaluated in terms of the D_mean_ and volumes or percent of volumes receiving different dose level (Vx, x = the interested dose level). For clarity, the Vx and Vx represented the absolute and relative volumes, respectively. The absolute volumes of V15, V30, V_40_ and V_50_ for small bowel and V_10_, V_20_, V_30_, V_40_ and V_50_ for normal tissue were reported. The percentages of total volume of V_30_, V_40_ and V_50_ for bladder and V_30_ and V_40_ for femoral heads were also investigated. The normal tissue was defined as the whole body excludes the PTV-C.

For statistical analysis, all dosimetric results from different irradiation techniques were compared with each other. The paired, two-tailed Wilcoxon signed-rank test was applied. Results were considered statistically significant with *p* < 0.05.
